# Pathogenomics and Evolutionary Epidemiology of Multi-Drug Resistant Clinical *Klebsiella pneumoniae* Isolated from Pretoria, South Africa

**DOI:** 10.1038/s41598-020-58012-8

**Published:** 2020-01-27

**Authors:** Nontombi Marylucy Mbelle, Charles Feldman, John Osei Sekyere, Nontuthuko Excellent Maningi, Lesedi Modipane, Sabiha Yusuf Essack

**Affiliations:** 1 Department of Medical Microbiology, Faculty of Health Sciences, University of Pretoria, Pretoria, South Africa; 2 National Health Laboratory Service, Johannesburg, South Africa; 3 Department of Internal Medicine, Faculty of Health Sciences, University of the Witwatersrand, Johannesburg, South Africa; 4 Antimicrobial Research Unit, College of Health Sciences, University of KwaZulu/Natal, Durban, South Africa

**Keywords:** Antimicrobial resistance, Bacteriology, Clinical microbiology, Bacterial genetics, Epidemiology

## Abstract

Antibiotic-resistant *Klebsiella pneumoniae* is increasingly being implicated in invasive infections worldwide with high mortalities. Forty-two multidrug resistant (MDR) *K. pneumoniae* isolates were collected over a 4-month period. Antimicrobial susceptibility was determined using Microscan. The evolutionary epidemiology, resistome, virulome and mobilome of the isolates were characterised using whole-genome sequencing and bioinformatics analysis. All isolates contained the *bla*_CTX-M_ gene, whilst 41/42(97%) contained *bla*_TEM_, 36/42(86%) contained *bla*_OXA_ and 35/42(83%) harboured *bla*_SHV_ genes. Other resistance genes found included *bla*_LEN_, *aac(6*′*)-lb-cr*, *qnr*A, *qnr*B, *qnr*S, *oqx*AB, *aad*, *aph*, *dfr*, *sul*1, *sul*2, *fos*A, and *cat* genes. Fluoroquinolone and colistin resistance-conferring mutations in *par*C, *gyr*AB, *pmr*AB, *pho*PQ and *kpn*EF were identified. The *bla*_LEN_ gene, rarely described worldwide, was identified in four isolates. The isolates comprised diverse sequence types, the most common being ST152 in 7/42(17%) isolates; clone-specific O and K capsule types were identified. Diverse virulence genes that were not clone-specific were identified in all but one isolate. IncF, IncH and IncI plasmid replicons and two novel integrons were present. The *bla*_CTX-M-15_ and *bla*_TEM-1_ genes were bracketed by Tn3 transposons, IS*Ec9*, a resolvase and IS91 insertion sequence. There were 20 gene cassettes in 14 different cassette arrays, with the *dfrA* and *aadA* gene cassettes being the most frequent. Phylogenetic analysis demonstrated that the isolates were evolutionarily associated with strains from both South Africa and abroad. These findings depict the rich resistome, mobilome and virulome repertoire in clinical *K. pneumoniae* strains, which are mainly transmitted by clonal, multiclonal and horizontal means in South Africa.

## Introduction

Antibiotic resistance (ABR) is a global phenomenon widely described in the literature^1,2^, and is associated with treatment failure, as well as increased morbidity and mortality^3–5^. Dissemination of resistance in bacteria, particularly among *Enterobacteriaceae*, is mainly due to the exchange of ABR genes (ARGs) between and within species, mediated by mobile genetic elements (MGEs) harbouring ARGs^6–8^. MGEs include plasmids, transposons and integrons that are known to transmit ABR in both Gram-positive and Gram-negative bacteria, including cephalosporin- and carbapenem-resistant *Enterobacteriaceae* that have been classified as critical priority pathogens by the WHO^9^. Integrons that capture cassettes i.e., single gene fragments, usually insert into transposons, enabling their movement between bacteria. Of the eight integron classes described, class 1, 2 and 3 are associated with antimicrobial resistance although class 1 is more frequently described in the literature^6,10–12^.

Among *Enterobacteriaceae*, *Klebsiella pneumoniae* is increasingly implicated as an invasive and virulent pathogen that harbours several ARGs^13^, including AmpCs, extended-spectrum β-lactamases (ESBLs) and carbapenemases. Cephalosporin- and carbapenem-resistant *K. pneumoniae* have been reported in South Africa to cause several mortalities in Johannesburg (Gauteng Province), Cape Town (Western Cape Province) and KwaZulu-Natal Province in South Africa^7,14–17^, including fatal outbreaks among infants^18^. We subsequently undertook a four-month molecular surveillance of a referral laboratory that serves two major tertiary hospitals in Pretoria, South Africa, to determine the evolutionary epidemiology, resistance mechanisms and associated MGEs in antibiotic-resistant *K. pneumoniae* isolates.

## Methods

### Study design

The study sample consisted of 42 multidrug-resistant (MDR) *K. pneumoniae* isolates that were collected as part of a larger study where consecutive ESBL-positive *Enterobacteriaceae*, co-resistant to fluoroquinolones and aminoglycosides, were surveilled^8,12,19^. The isolates were collected from a referral laboratory of the National Health Laboratory Services (NHLS), in Pretoria, South Africa.

### Bacterial isolates

**Figure 1:**
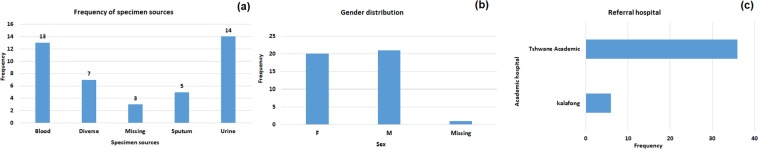
Demographic characteristics of patients from whom the clinical specimen were obtained. The patients were of almost equal distribution in terms of sex although their ages varied widely. The samples were obtained mainly from Steve Biko Academic Hospital and Kalafong.

These 42 ESBL-producing *K. pneumoniae* clinical isolates were identified from urine (n = 14), blood (n = 13), pus/pus swab and other sources (n = 7), sputum (n = 5) and missing (n = 3) specimens of patients admitted to two referral hospitals over a four-month period from September to December in 2013 (Fig. 1). All the samples were collected from infected patients and plated on blood agar (Oxoid, Basingstoke, UK) for 24 hours at 37 °C. They were re-plated unto Mueller-Hinton agar for antibiotic sensitivity testing and ESBL screening using the disc diffusion method: cefoxitin, ceftazidime, and clavulanic acid antibiotic discs^20^.

### Identification and antimicrobial susceptibility testing

The MicroScan WalkWay7465 (Beckman Coulter, Sacramento, USA) instrument was used to identify the species and establish the susceptibility of the isolates to 32 antibiotics: penicillins, cephems, carbapenems, polymyxins, fluoroquinolones, aminoglycosides, tetracyclines, tigecycline, sulphamethoxazole-trimethoprim, nitrofurantoin and fosfomycin (Table S1). The CLSI guidelines were used to interpret the minimum inhibitory concentrations (MICs) (CLSI M100) except for colistin and tigecycline for which the EUCAST breakpoints were used^21,22^. The taxonomic identity of the isolates was confirmed with the average nucleotide identity (ANI) database of NCBI.

### Whole-genome sequencing

Genomic DNA was extracted from the isolates using the ZR Fungal/Bacterial DNA Mini-Prep kit (Zymo Research, Epigenetics, USA). Whole-genome sequencing was performed on the Ion torrent using already described methods^23,24^. Briefly, the genomic DNA were sheared to 200-bp libraries; 280-bp fragments were selected using 2% agarose gels and Pippen prep (Sage Science, Beverly, MA, USA). Individual libraries were pooled and sequenced on the Ion Proton (ThermoFisher, Waltham, MA, USA). The generated raw reads were de novo assembled using the SPAdes assembler.

### Analysis of whole genome sequence data

Assembled sequences were annotated using the ResFinder web server and PGAP to detect resistance genes^25,26^. The MLST sequence types were identified on the MLST database hosted by the CGE (http://cge.cbs.dtu.dk/services/MLST/). The integrons and gene cassettes within the genomes were identified according to the INTEGRALL database (http://integrall.bio.ua.pt/). Plasmid types were identified using PlasmidFinder on the CGE (http://cge.cbs.dtu.dk/services/PlasmidFinder/) website. The capsule types were identified using Kaptive Web whilst the virulome was characterised using BacWGSTdb^27,28^. All sequences were deposited at the GenBank data-base and have been allocated accession numbers (Supplementary Tables S1 and S2) under Bioproject PRJNA355910.

### Phylogenomic analysis

Whole genome sequences (WGS) from this study were analysed alongside whole-genome sequences of *K. pneumoniae* isolates curated at the PATRIC website (https.www.patric.org), from where genomes sequenced between 2000 and 2019 were downloaded to enable a current epidemiological and evolutionary analysis; WGS sequences of isolates from Durban, KwaZulu Natal Province, at Genbank (PRJNA287968) were also included. The downloaded sequences (n = 700 isolates) were analysed by RAxML and Parsnp, which was used for the phylogenetic analysis, using the “-C 1000 –c” flag to enable alignment over collinear regions^29^. The engendered trees were viewed with Gingr (https://harvest.readthedocs.io/en/latest/content/gingr.html) and edited with Figtree (http://tree.bio.ed.ac.uk/software/figtree/).

## Results

### Patient details

Most of the isolates (n = 36, 86%) were from patients admitted at the Tshwane Academic referral hospital and the rest were from Kalafong. Twenty of the patients were males whilst 22 were females, with ages ranging between 2 and 89 years old and a mean age of 39 years. Most of the samples were either from blood or urine, with five being from sputum (Fig. 1; Supplementary Table S1).

### Genomic characteristics

The draft genome sizes of the isolates ranged from 5.2 to 5.7 Mb, with very diverse L50, N50 and contig numbers. The CRISPR arrays in the isolates ranged from zero (n = 21 isolates) to four (n = 1 isolate) (Supplementary Table S1).

### Antibiotic susceptibility of isolates

**Figure 2:**
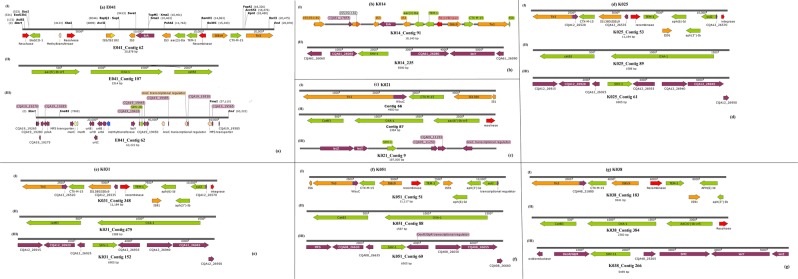
Genetic environment of β-lactamases found in the *Klebsiella pneumoniae* strains. The genetic environment of the ESBL genes viz., *bla*_CTX-M-15_, *bla*_TEM-1B_, *bla*_OXA-1/9_, and *bla*_SHV_ were determined using the annotated GFF files from GenBank. The *bla*_SHV_ genes were mostly found on chromosomes whilst the *bla*_CTX-M-15_ and *bla*_TEM-1B_, genes were mostly found on Tn3 transposons, ISEc9 and IS91.

**Figure 3:**
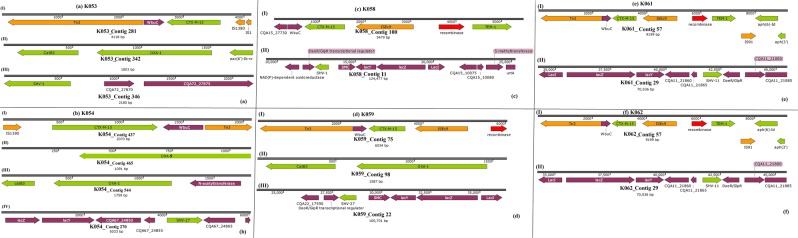
Genetic environment of β-lactamases found in the *Klebsiella pneumoniae* strains. The genetic environment of the ESBL genes viz., *bla*_CTX-M-15_, *bla*_TEM-1B_, *bla*_OXA-1/9_, and *bla*_SHV_ were determined using the annotated GFF files from GenBank. The *bla*_SHV_ genes were mostly found on chromosomes whilst the *bla*_CTX-M-15_ and *bla*_TEM-1B_, genes were mostly found on Tn3 transposons, ISEc9 and IS91.

**Figure 4:**
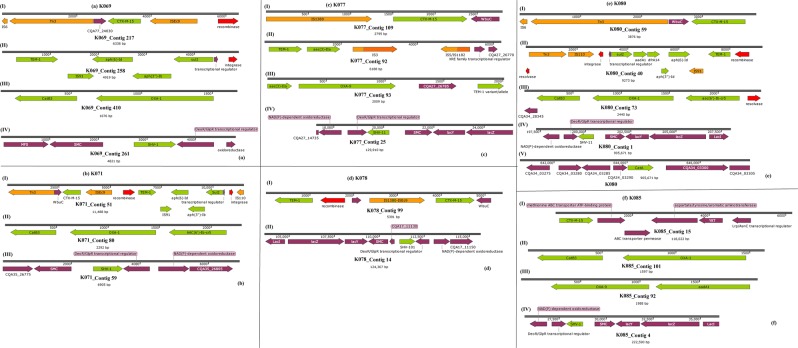
Genetic environment of β-lactamases found in the *Klebsiella pneumoniae* strains. The genetic environment of the ESBL genes viz., *bla*_CTX-M-15_, *bla*_TEM-1B_, *bla*_OXA-1/9_, and *bla*_SHV_ were determined using the annotated GFF files from GenBank. The *bla*_SHV_ genes were mostly found on chromosomes whilst the *bla*_CTX-M-15_ and *bla*_TEM-1B_, genes were mostly found on Tn3 transposons, ISEc9 and IS91.

**Figure 5:**
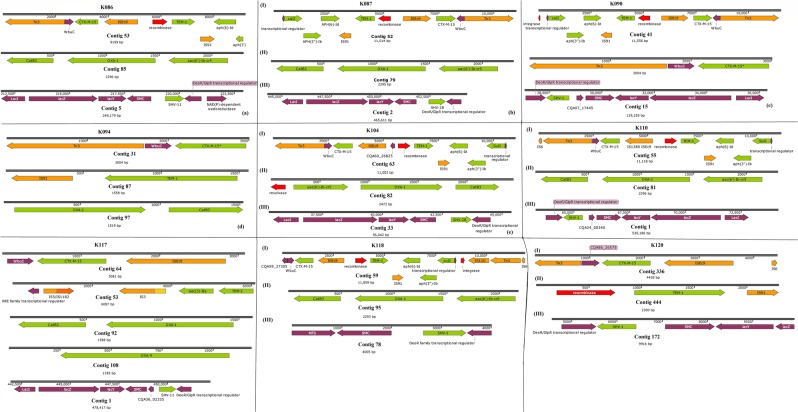
Genetic environment of β-lactamases found in the *Klebsiella pneumoniae* strains. The genetic environment of the ESBL genes viz., *bla*_CTX-M-15_, *bla*_TEM-1B_, *bla*_OXA-1/9_, and *bla*_SHV_ were determined using the annotated GFF files from GenBank. The *bla*_SHV_ genes were mostly found on chromosomes whilst the *bla*_CTX-M-15_ and *bla*_TEM-1B_, genes were mostly found on Tn3 transposons, ISEc9 and IS91.

**Figure 6:**
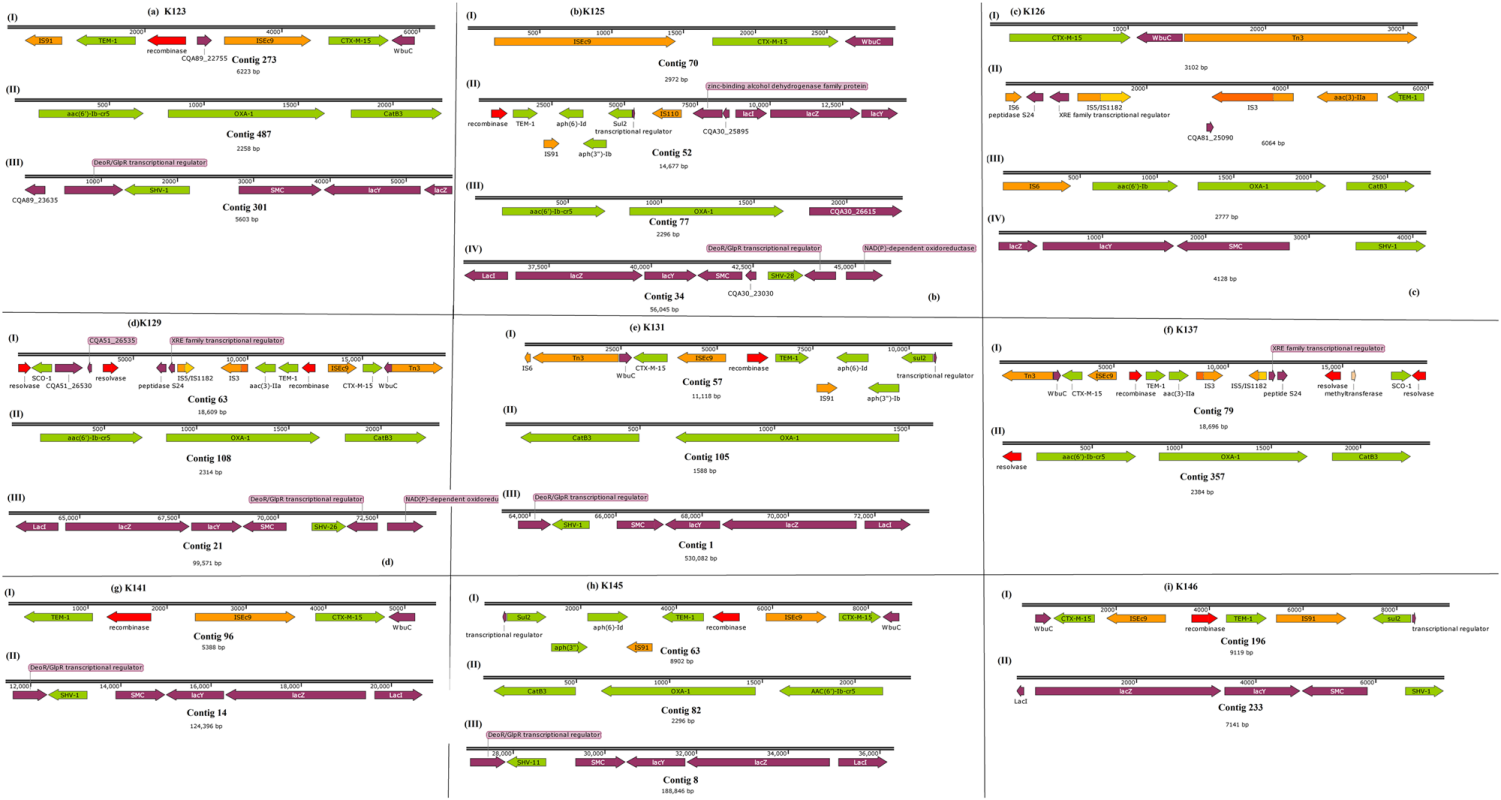
Genetic environment of β-lactamases found in the *Klebsiella pneumoniae* strains. The genetic environment of the ESBL genes viz., *bla*_CTX-M-15_, *bla*_TEM-1B_, *bla*_OXA-1/9_, and *bla*_SHV_ were determined using the annotated GFF files from GenBank. The *bla*_SHV_ genes were mostly found on chromosomes whilst the *bla*_CTX-M-15_ and *bla*_TEM-1B_, genes were mostly found on Tn3 transposons, ISEc9 and IS91.

**Figure 7:**
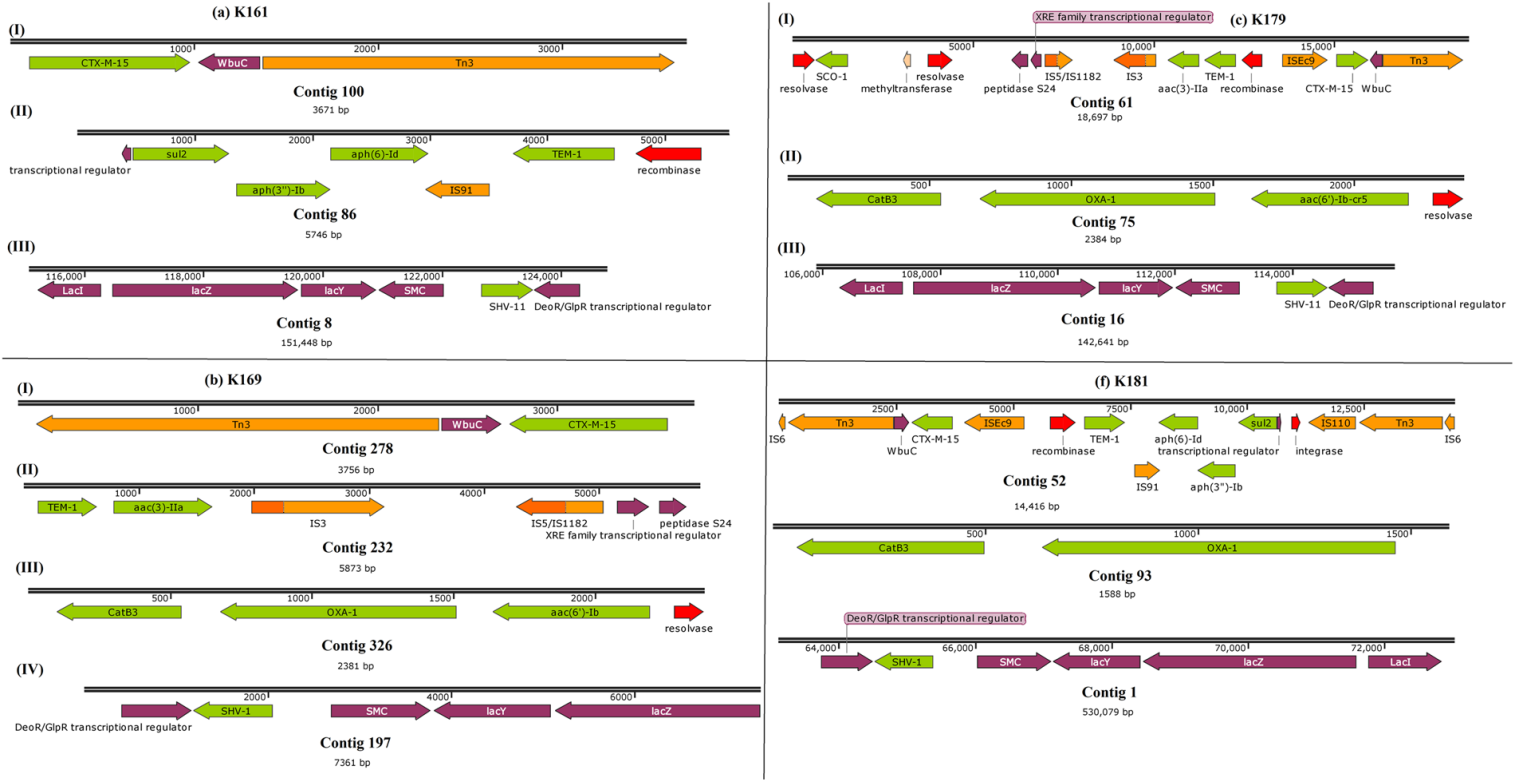
Genetic environment of β-lactamases found in the *Klebsiella pneumoniae* strains. The genetic environment of the ESBL genes viz., *bla*_CTX-M-15_, *bla*_TEM-1B_, *bla*_OXA-1/9_, and *bla*_SHV_ were determined using the annotated GFF files from GenBank. The *bla*_SHV_ genes were mostly found on chromosomes whilst the *bla*_CTX-M-15_ and *bla*_TEM-1B_, genes were mostly found on Tn3 transposons, ISEc9 and IS91.

The isolates obtained from the samples were identified phenotypically and genomically as *K. pneumoniae*. All the isolates were phenotypically positive for extended-spectrum β-lactamase (ESBLs) production. They were mostly resistant or non-susceptible to all the β-lactams except the carbapenems and cephem-β-lactamase inhibitors viz., sulbactam, tazobactam and clavulanic acid. Specifically, all isolates were resistant to aztreonam, cephalothin, cefuroxime, cefotaxime, ceftazidime and cefepime. Notably, resistance to amoxicillin-clavulanate (AUG) and piperacillin-tazobactam was very common compared to cephem-β-lactamase inhibitor combinations. Sixteen isolates had cefoxitin MICs ≥ 16 mg/L. There was variable susceptibility to carbapenems with all isolates demonstrating MICs ≤ 1 mg/L for imipenem and meropenem. Overall, 41 isolates had a doripenem MIC ≤ 1 mg/L, and four isolates, namely K181, K145, K059 and K091, had ertapenem MICs of >1 mg/L (Supplementary Table S2). The phenotypic resistance data tallied largely with the genomic results in that there was no carbapenemase gene found, confirming the absence of carbapenem resistance. As well, the phenotypic ESBL results were confirmed by the presence of *bla*_TEM_, *bla*_CTX-M-15_, *bla*_SHV_*, bla*_OXA_ and *bla*_LEN_ genes (Tables 1–2, Figs. 2–7 and supplementary Table S1).

**Table 1:** Sequence types (ST), antibiotic resistance genes and integrons found in the *Klebsiella pneumoniae* isolates.

Sample code (MLST)	Integron	Cassette arrays					
		GC1	GC2	GC3	GC4	GC5	GC6
K021 (ST152)	ln1481*	aadA16	—	—	—	—	—
K058 (ST1414)	ln191, ln792, ln54	dfrA17 dfrA14b aadA16 aacA4	aadA5 arr3	—	—	—	—
K089 (ST1552)	ln369	dfrA1d	—	—	—	—	—
K179 (ST39)	ln388	dfrA15	aadA1a	—	—	—	—
E041 (ST15)	ln388	dfrA15	dfrA15	—	—	—	—
K025 (ST152)	ln369	dfrA1b	aadA1a	—	—	—	—
K031 (ST152)	ln18	dfrA1b	aadA1a	—	—	—	—
K051 (ST152)	ln369	dfrA17	aadA5	—	—	—	—
K059 (ST234)	ln191, ln27	dfrA14b dfrA12	gcuF	aadA2	—	—	—
K061 (ST1552)	ln369	dfrA1b	aadA1b	—	—	—	—
K078 (ST1414)	ln191, ln792, ln54	dfrA1b aacA4 dfrA17	Arr3 aadA5	—	—	—	—
K086 (ST1552)	ln369	dfrA1b	aadA1b	—	—	—	—
K094	ln191	dfrA1b	—	—	—	—	—
K090 (ST323)	ln191	dfrA1b	—	—	—	—	—
K087 (ST15)	ln191	dfrA1b	—	—	—	—	—
K110 (ST152)	ln369	dfrA1b	aadA1b	—	—	—	—
K117 (ST1552)	None	dfrA1b	aadA1	—	—	—	—
K141 (ST1414)	ln191, ln54	dfrA17	aadA5	—	—	—	—
K077 (ST1552)	—	dfrA1b	aadA1	—	—	—	—
K071 (ST152)	ln369	dfrA1b dfrA14b	aadA1b	—	—	—	—
K080 (ST234)	ln369	dfrA1b	aadA1b	—	—	—	—
K181 (ST152)	ln0		—	—	—	—	—
K125 (ST15)	ln191	dfrA1b	—	—	—	—	—
K145 (ST39)	ln191, ln1482*	dfrA1b dfrA30b	—	—	—	—	—
K161 (ST25)	ln1229	aacA4cr	arr3	dfrA27	—	—	—
K085 (ST101)	ln191	dfrA14b	—	—	—	—	—
K104 (ST15)	ln388	dfrA15	aadA1a	—	—	—	—
K129 (ST643)	ln388	dfrA15	aadA1a	—	—	—	—
K131 (ST152)	None	aacA4cr	—	—	—	—	—
K118 (ST14)	ln191, ln22	dfrA7 dfrA14b	—	—	—	—	—
K014 (ST607)	None	aacA4cr	—	—	—	—	—
K038 (ST1552)	ln369	dfrA14b dfrA1b	aadA1b	—	—	—	—
K053 (ST234)	ln191	dfrA14b	—	—	—	—	—
K054 (ST234)	In27	dfrA12 dfrA14b	gcuF	aadA2	—	—	—
K062 (ST234)	In27	dfrA12	gcuF	aadA2	—	—	—
K069 (ST152)	In369	dfrA1b aacA4cr	aadA1b	—	—	—	—
K120 (ST17)	None	dfrA14b	—	—	—	—	—
K123	In191	dfrA14b	—	—	—	—	—
K126 (ST101)	In369	dfrA14b	aadA1b	—	—	—	—
K137 (ST182)	In388	dfrA15	aadA1a	—	—	—	—
K146 (1414)	In191	dfrA14b	—	—	—	—	—
	ln792	aacA4	arr3		—	—	—
	In54	dfrA17	aadA5		—	—	—
K169 (ST101)	In369	dfrA1b	aadA1b	—	—	—	—

^*^New integrons are identified by an asterix.

**Table 2:** Point mutations in the *parCE* and *gyrAB* genes of the *Klebsiella pneumoniae* isolates from South Africa.

Isolate ID	Genes^*^			
	*gyrA*	*gyrB*	*ParC*	*ParE*
E041	—	—	—	—
K104	S83F, D87A, N645H	—	S80I	—
K110	S83F, D87A	—	S80I	—
K117	—	—	—	—
K118	—	—	—	—
K120	—	—	—	—
K123	—	—	—	—
K125	S83F, D87A	—	S80I	—
K126	—	D553V	S80I, N304S	—
K129	—	L657M	H364N	—
K131	S83F, D87A	—	S80I	—
K137	—	—	D397E	—
K014	—	—	—	—
K141	—	—	E637A	—
K145	—	—	—	—
K146	—	—	E637A	—
K161	—	—	—	—
K169	S83Y, D87G	D553V	S80I	—
K181	S83F, D87A	—	S80I	—
K021	S83F, D87A	—	S80I	—
K025	S83F, D87A	—	S80I	—
K031	S83F, D87A	—	S80I	—
K038	—	—	—	—
K051	S83F, D87A	—	S80I	—
K053	—	—	—	—
K054	—	—	—	—
K058	—	—	E637A	—
K059	—	—	—	—
K061	—	—	—	—
K062	—	—	—	—
K069	—	—	S80I	—
K071	S83F, D87A	—	S80I	—
K077	—	—	—	—
K078	—	—	E637A	—
K080	—	—	—	—
K085	S83Y, D87G	D553V	S80I, N304S	—
K086	—	—	—	—
K087	S83F, D87A	—	S80I	—
K089	—	—	—	—
K094	—	—	—	—
K090	—	—	N304S	—
K179/K0179	—	—	—	—

*Reference *K. pneumoniae* genome used was *K. pneumoniae* ATCC 13883 (PRJNA244567).

Except for amikacin, the isolates were resistant to all aminoglycosides. Further, all the isolates were resistant to ciprofloxacin but susceptible to norfloxacin; twenty and two were respectively resistant to levofloxacin and nalidixic acid (Supplementary Table S2). Resistance to minocycline (n = 18 isolates) and tetracycline (n = 25 isolates) was common than tigecycline (n = 7 isolates). Resistance to chloramphenicol (n = 33), colistin (n = 10), nitrofurantoin (n = 16) and sulphamethoxazole-trimethoprim (SXT) (n = 42) was identified, with none being resistant to Fosfomycin (Supplementary Table S2). Contrary to the β-lactams, there were substantial discrepancies between the phenotypic and genomic results for the non-β-lactam antibiotics. For instance, the isolates were mostly susceptible to Fosfomycin although *fosA* genes were ubiquitous among the isolates. Further, the presence of *aac(6*′*)-Ib, OqxAB, Qnr* and *aac(3*′*)-Ib* genes and mutations in *parC* and *gyrAB* only conferred resistance to ciprofloxacin and levofloxacin but not to norfloxacin. A similar observation was made with regards to amikacin and gentamicin/tobramycin (*aadA4, aph(3*′*), strAB, aac(6*′*)-Ib-cr*) and between minocycline, tetracycline and tigecycline (*tet*). However, the presence of chloramphenicol and SXT resistance was mostly corroborated by the presence of the appropriate resistance genes i.e., *cml/Cat* and *sul/dfrA*. For some strains and antibiotics, no known resistance determinant was found to explain the observed phenotypic resistance (Supplementary Tables S1 and S2).

### ARGs in Klebsiella pneumoniae

Several resistance genes were present in the isolates (Table 2 and Supplementary Table S1). Many isolates had at least two β-lactamase genes, with *bla*_CTX-M-15_ (n = 42), *bla*_*OXA*_ and *bla*_TEM_ being commonest (Figs. 2–7). The *bla*_TEM_ gene was present in 41/42 (97.6%) isolates, *bla*_OXA_ was present in 36/42 (85.7%) isolates and *bla*_SHV_ in 35/42 (83.3%). We identified three *bla*_TEM_ genes, of which the most frequent was *bla*_TEM-1B._ Isolate K021 did not have a TEM β-lactamase but had the *bla*_OXA_ and *bla*_CTX-M-15_ genes. WGS revealed several *bla*_SHV_ and *bla*_OXA_ genes, with the most diverse being found in the *bla*_*S*HV_-containing isolates. The *bla*_LEN_ gene, first identified by Arakawa *et al*. in 1986^30^, was detected in four isolates, namely K053, K126, K137 and K146.

The *Qnr*, *Oqx* and *aac(6*′*)-lb-cr* plasmid-mediated quinolone resistance (PMQR) genes were detected in fluoroquinolone-resistant isolates. Of the isolates with the *qnr* gene, the *qnr*B fluoroquinolone resistance gene was the most frequently identified (20/42, 47.6%). Both the *qnr*B6 and *qnr*B66 genes were identified in these isolates. Six out of 42 (14.3%) and 5/42 (11.9%) isolates had the *qnr*A and *qnr*S genes, respectively. The *oqx*A and *oqx*B genes were both identified in 36/42 (85.7%) isolates. The most frequently identified PMQR gene was the *aac(6*′*)-lb-cr* gene, which was detected in 38/42 (90%) isolates. Six other *aac(6*′*)-lb* genes were identified. Twenty-one (50%) isolates had mutations in the chromosomally encoded *parC, gyrA* and *gyrB* quinolone resistance-determining region (QRDR) genes (Table 3).

**Table 3:** Point mutations in the colistin chromosomal resistance genes of the *Klebsiella pneumoniae* isolates from South Africa.

Isolate ID	MIC	*pmr*B	*pmr*A	*pho*P	*pho*Q	*kpn*E	*kpn*F	*mgr*B	*ccrB*
K118	>4	A246T, L339C, H340I, N341T, R342D, Q343S, P346Q	—	—	—	K112Q	—	—	Gene not found
K141	>4	—	—	—	—	—	—	—	Gene not found
K145	4	—	M66I	—	—	L7^*^	—	—	
K146	4	—	—	—	—	—	—	—	
K021	4	—	—	—	S405R, H406Y	—	—	—	
K031	4	—	—	—	H406Y	—	—	—	
K053	4	A246T	S64T	—	—	—	—	—	
K054	4	A246T	S64T	—	—	—	—	—	
K059	4	A246T	S64T	—	—	—	—	—	
K085	4	—	A217V	—	—	—	I39F, Del RRKIYGF (40–46)	—	
K090	4	—	—	—	—	I88M, K107R	Y66T	—	C12Y, A35V, Y36H, G191E, N388D, S379P, W404R
K038	≤2	—	—	—	—	—	—	—	C12Y, G191E, S379P, 404R, F213L, T248I, Q258R
K080	≤2	—	—	—	—	—	—	—	
K077	≤2	—	—	—	—	—	—	—	
K117	≤2	—	—	—	—	—	—	—	
K086	≤2	—	—	—	—	—	—	—	
K061	≤2	—	—	—	—	—	—	—	
K089	≤2	—	—	—	—	—	—	—	

^*^Reference *K. pneumoniae* genome used was *K. pneumoniae* ATCC 13883 (PRJNA244567).

The *aac, aad* and *aph* aminoglycoside-modifying enzyme genes were detected in all isolates: several *aac* genes such as *aac*(3′)−11a (35/42, 83.3%), *aac*(3)−11d (4/42, 9.5%), *aac* (6′)−1b (6/42, 14.3%) and *aac*A4 (10/42, 23.8%) were found. We identified *aad* modifying enzymes, including *aad*A1 in 26/42 (61.9%) isolates, *aad*A2 in 3/42(7.1%) isolates, *aad*A5 in 4/42 (9.5%) isolates and *aad*A16 in 3/42 (7.1%) isolates. The *aph*(3′)−1a gene was detected in 7/42 (16.7%) isolates. Streptomycin resistance genes, *str*A and *strB* were found in 35/42 (83%) and 34/42 (81%) isolates, respectively (Table 1).

The *fos*A gene, which codes for fosfomycin resistance was identified in 40/42 (95%) isolates. No other *fos* gene was identified. Trimethoprim–sulfamethoxazole resistance was encoded by *sul* and *dfr* genes. We identified *sul*1 in 33/42 (78%) isolates and *sul*2 in 36/42 (86%) isolates. We identified several *dfr* genes, including *dfr*A4 in 15/42 (36%) isolates, *dfr*A1 in 14/42 (33%) isolates, *dfr*A27 in 8/42 (19%) isolates, *dfr*A15 in 6/42 (14%) isolates, *dfr*A12 in 3/42 (7%) isolates, *dfr*A7 in 1/42 (2%) isolates and *dfr*A30 in 1/42 (2%) isolate. Both the fosfomycin and trimethoprim-sulfamethoxazole genes were co-carried together with the β-lactamase, aminoglycoside and fluoroquinolone resistance genes (Figs. 2–7; Table 1; Supplementary Table S2).

No plasmid-mediated colistin resistance gene was identified in the isolates having increased colistin MICs. Chromosomally encoded mutations in the *pmr, pho* and *kpn* genes were, however, identified in 9/11 (82%) of these isolates (Table 1). No novel putative colistin ARGs were identified on any of the available databases. Isolates with *ccrB* mutations, however, were susceptible to colistin and no truncation were observed in the *mgrB* in all the isolates (Table 3).

Two isolates contained two different exporter, efflux pump genes for chloramphenicol. Isolate K120 had the *flo*R gene and isolate K021 had the *cml*A1 gene. Several chloramphenicol acetyltransferase genes were also identified: *cat*B3 in 34/42 (81%) isolates, *cat*A1 in 23/42 (55%) isolates and *cat*A2 in 8/42 (19%) isolates. Isolates with chloramphenicol resistance had one or more transferase genes. The rifampicin ADP ribosylating transferase *ARR*−3 gene was detected in 12/42 (29%) isolates. Moreover, the ARGs were not influenced by the isolation source of the isolates as isolates from blood, urine; in fact, in some cases isolates from sputum had more ARGs than those from urine or blood (Supplementary Table S1).

### Sequence types and the genetic environment of the ARGs

We detected 11 different sequence types in the isolates (Table 3). The most prevalent sequence types were the ST152 (n = 9, 33%) and ST1552 (n = 6, 22%). Both sequence types were associated with integron *ln*369 (Table 1). The globally distributed ST15 was only detected in four isolates (Tables 1–2).

As well, we identified the IncF, IncN and IncH incompatibility plasmid replicons (Supplementary Table 1), with nine isolates carrying multiple plasmid replicons simultaneously. The IncF plasmid group was most frequently identified. Nine of the isolates with this IncF incompatibility group also harboured the IncH group. Several unknown plasmid sequence types were also identified (Table 3). The IncF group was also associated with most of the STs.

All isolates contained only class 1 integrons. Several isolates had multiple integrons; K058, K025, K031, K051, K059, K061, K078, K086, K080, K145, K118, K038 K054 and K117. These integrons circulated between both tertiary hospitals (Supplementary Table S2). Two novel class 1 integrons were identified in two isolates, K021 and K145, which were given new numbers, In1481 and ln1482, respectively. The isolates were registered in the GeneBank database with specific accession numbers NXIU000108 (In1481) and NXKB01000066 (ln1482).

The most frequent integron on the IncF plasmid was In369, which captured the *dfr*A1b – *aad*A1b cassette array and has been described in other *Enterobacteriaceae*. Two isolates also harboured the *aac*A4cr cassette gene. This integron was more frequently associated with ST1552 and ST152 but was also found in two ST101 isolates and one ST234 isolate. In27, a narrow spectrum integron that has been described in the literature was only identified in ST234 isolates capturing the *dfr*A12-*gcu*F–*aad*A2 cassette array, confirming its narrow spectrum status. In191 was, however, identified in four different sequence types, indicating that it is not a narrow spectrum integron.

Most isolates had gene cassettes associated with trimethoprim and aminoglycoside resistance (Table 3). The frequency, in descending order, was *dfrA* in 38/42 (90%) isolates, *aadA* in 25/42 (59%) isolates, *aacA* and *arr* in 4/42 (9%) isolates and *gcu* in 3/42 (7%) isolates. Ten different *dfr* gene cassettes were identified, the most common being *dfrA1b* (n = 20), *dfrA14b* (n = 9) and *dfrA15* (n = 6). Of the six *aadA* cassettes identified, the most frequent were *aadA1b* (n = 8) and *aadA1a* (n = 6). Only two *aacA* cassettes were identified, *aacA4cr* (n = 3) and *aacA4* (n = 2). A *gcu* gene cassette of unknown function was identified in three isolates. The most frequent gene cassette array was the *dfrA1b*-*aadA1b* cassette array, responsible for trimethoprim and streptomycin resistance. No β–lactamase cassette was captured in any of the isolates.

Except for *bla*_SHV_ genes, which were mostly chromosomal and not associated with any transposon, integron (resolvase/recombinase) and insertion sequence (IS), the *bla*_TEM_, *bla*_CTX-M-15_ and *bla*_OXA_ genes were mostly bracketed by mobile genetic elements. In particular, *bla*_TEM_ and *bla*_CTX-M-15_ genes co-existed within composite Tn3 transposons, with the *bla*_CTX-M-15_ being directly joined to an ISEc9 IS; *bla*_TEM_ was mostly bracketed by a resolvase, an IS91 and *aph(6)-Id, aph(*3′*)-Ib*, and *sul2*. The genetic context of *bla*_CTX-M-15_ in K085 strongly suggests its presence on the chromosome (Fig. 4f). The *bla*_OXA_ genes were also mostly associated with *aac(6*′*)-Ib-cr* and *cat* genes as well as a resolvase/recombinase (integrase). Notably, these associations were not clone specific as they occurred across different STs (Figs. 2–7).

### Virulome and capsular characteristics

**Figure 8:**
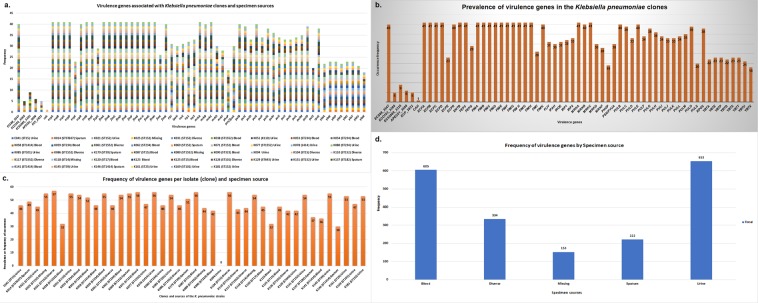
(**a–d**) Virulence genes distribution frequency and their association with specific *K. pneumoniae* clones and specimen sources. The virulence genes were not clone specific as same clones had different virulence genes (**a**). The virulence genes distribution and frequency were also not determined the specimen sources (**a**). K094 had no virulence gene, capping the highest frequency of virulence genes per isolate to 41 with the lowest (*cah*) being 1 (**b**). The highest number of virulence gene per clone was 57, with K094 having none (**c**). Strains from urine and blood had the highest number of virulence genes, which could obviously be due to the relatively larger number of strains isolated from urine and blood (**d**).

A total of 62 virulence genes were identified in all the strains, with *EC588_3547, ecpABCR, entB, fepC, fimABCDEFGHK, mrkABC, pulBCED* and *rpoS* occurring in almost all the isolates except for K094, which had no virulence gene (Fig. 8). Among the isolates, *EC0103_3368, EC55989_3335, APEC01_3698, ECP_2822* and *cah* were the least occurring, with *cah* being only present in K090. As shown in Fig. 8c, the virulome was not clone-specific in that isolates of the same clone had different virulence genes. Although most of these virulence genes occurred in isolates obtained from urine (n = 653) and blood (n = 605), their distribution does not suggest their association with these sources (Fig. 8). The highest number of virulence genes to occur in a single isolate was 57 (K031) whilst all but K094 had more than 30 virulence genes in a single isolate (Fig. 8c). hypervirulence genes were however absent.

**Figure 9:**
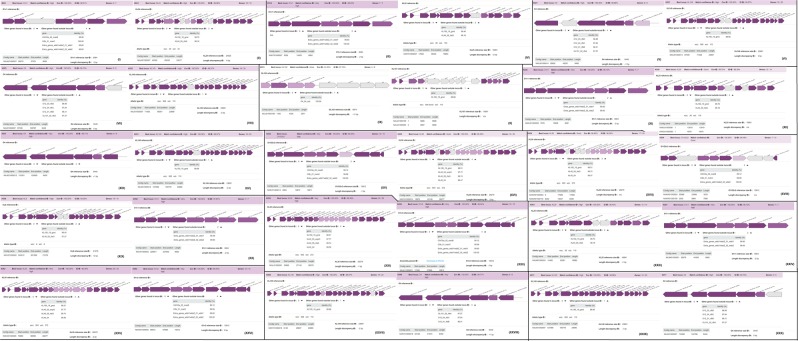
Types and distribution of O and K capsule types among the *K. pneumoniae* strains. Except for a few exceptions, the O and K capsule types were the same for isolates of the same clone (ST).

**Figure 10:**
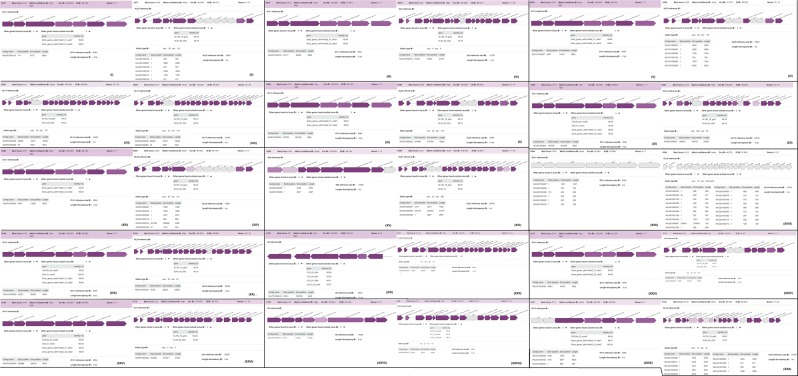
Types and distribution of O and K capsule types among the *K. pneumoniae* strains. Except for a few exceptions, the O and K capsule types were the same for isolates of the same clone (ST).

**Figure 11:**
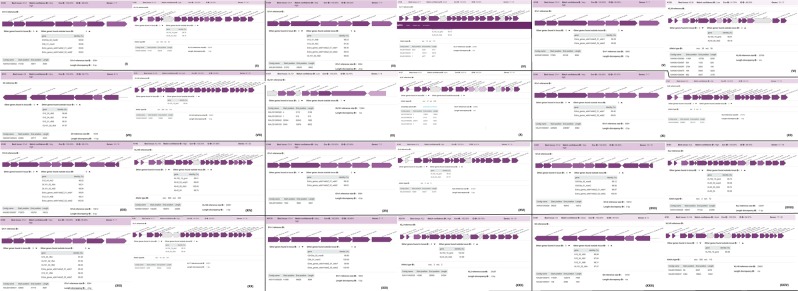
Types and distribution of O and K capsule types among the *K. pneumoniae* strains. Except for a few exceptions, the O and K capsule types were the same for isolates of the same clone (ST).

**Figure 12:**
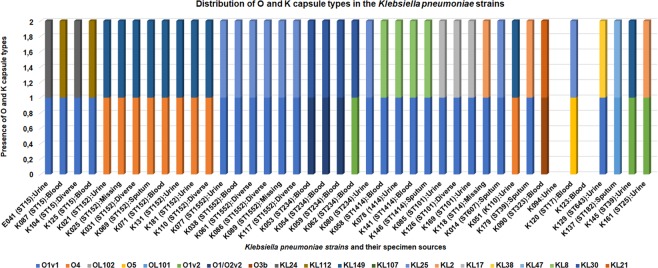
Types and distribution of O and K capsule types among the *K. pneumoniae* strains. Except for a few exceptions, the O and K capsule types were the same for isolates of the same clone (ST). O1v1 and KL149 were the most dominant capsule types, occurring across different clones (12).

The O and K capsules types in the strains were highly clone specific, with same clones having the same O and K capsule types. However, minor discrepancies were observed as some strains of the same clone had O and K capsule types that were different from those of members of the same clone. A case in point is that of K031 of ST152, which had OL102 and KL107 while other members of the same clone had O4 and KL149 (Figs. 9–12; Supplementary data S3). As can be observed, the O1v1 capsule type was more dominant across the clones whilst KL149 was the most dominant K capsule type. In all, there were eight different O capsule types whilst 12K capsule types were identified, evincing the higher diversity of the K capsule types. It is worthy of mention that isolate K094 had no O or K capsule, just as it also had no virulence genes (Figs. 9–12; Supplementary data 3), which is a very interesting finding

### Evolutionary phylogenomics and epidemiology

**Figure 13:**
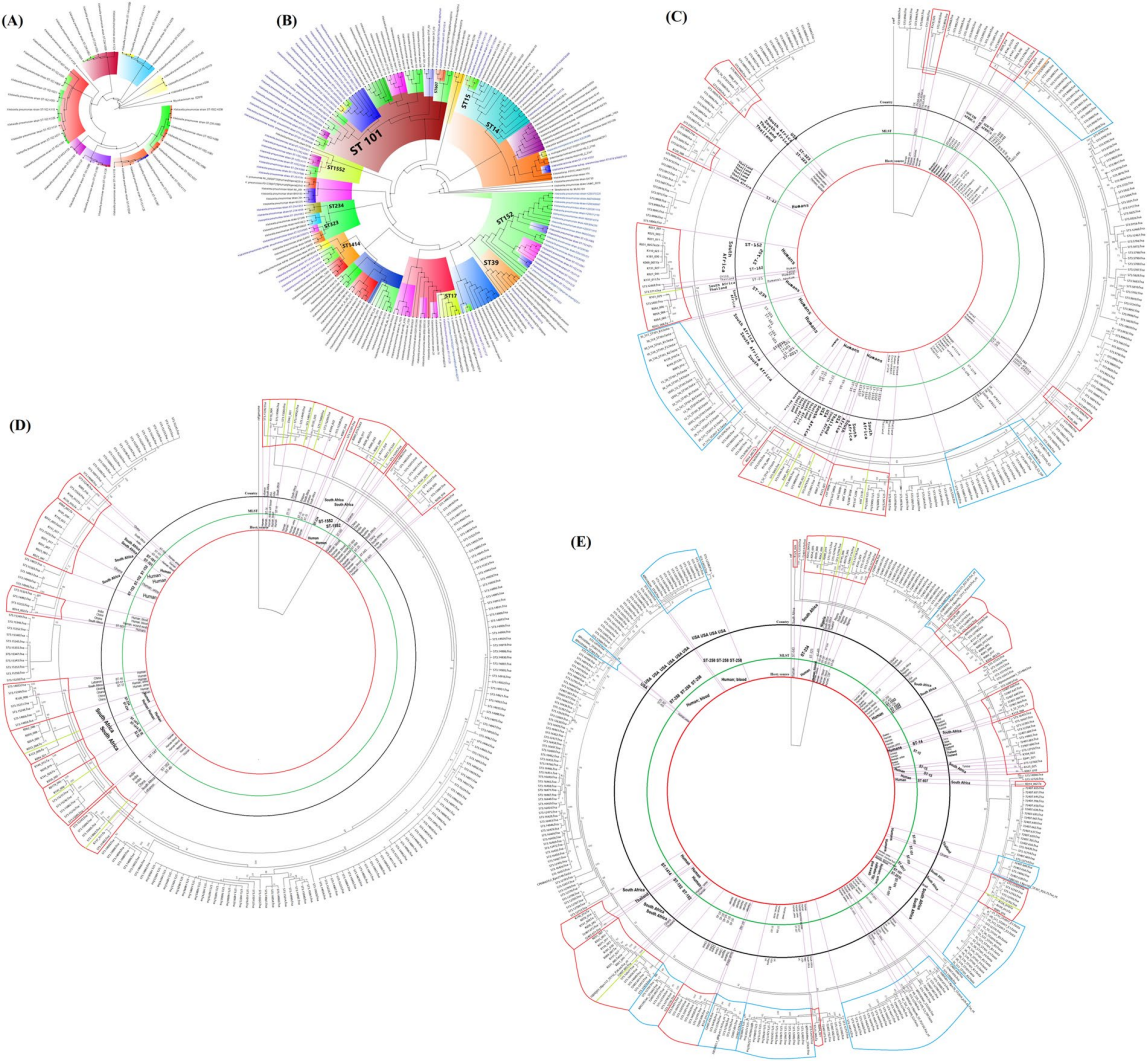
(**A–E**) Phylogenomic characterisation of the *K. pneumoniae* strains and their evolutionary relationship with African and International strains. Isolates of the same clone (ST) clustered together, although some strains of different STs were found within or close to strains of same STs (**A**). Isolates from South Africa are coloured in blue letters while those from this study have red branches. Strains from South Africa were largely clustering together albeit the isolates also clustered with strains from Nigeria, Cameroon, Uganda and Sudan that had the same or closely related ST. Notably, strains from Durban (South Africa) were more closely related to our strains (**B**). Globally, the strains were related to clones from Belgium, Brazil, China, Ghana, India, Lebanon, Thailand, UK, and USA (**C–E**). RAXmL and Parsnp were used to draw the trees, which were subsequently annotated with Figtree.

The *K. pneumoniae* isolates showed significant phylogenetic diversity (Fig. 13), with the whole-genome phylogenetics showing higher resolution than the MLST typing scheme. For instance, K080 (ST234) was phylogenetically closer to K038 (ST1552) than other ST1552 strains, which were themselves found on different branches, albeit of the same clade. Further, K129 (ST643) and K161 (ST25) were of very close evolutionary distance, albeit of the different clones and capsular types. However, strains of the same MLST clustered within the same clade, with some single STs clusteing closely. Examples include K118 (ST14) within the ST15 clade, K137 (ST182) within the ST101 clade, K014 (ST607) within the ST152 clade, K123 within the ST234 clade, and K090 (ST323) within the ST1414 clade. Interestingly, K120 (ST17), K001 (ST179) and K094 were phylogenetically distant from every strain within the collection (Fig. 13A).

Within the African context, the isolates (coloured with red branches and labelled in blue) were largely phylogenetically related to other *K. pneumoniae* strains of the same clones (STs) and clades such that strains of the same clones were clustered together within the same clade. This can be seen with K120 (ST17), which clustered with same clinical clones from Nigeria and South Africa; K0179 and K145 (ST39) with a ST38 strain from Nigeria, a ST39 strain from Cameroon and a strain from Uganda. This pattern is observed around the tree under the respective STs (Fig. 13B). However, as observed above, strains of different STs were also found clustered together; this can be seen with K137 (ST182) and K129 (ST643) and PR042E3 (ST31) from pigs in Cameroon (Fig. 13B; Supplementary data S4).

Globally, the STs clustered with same or closely related STs from Austria, Brazil, China, India, Lebanon, Thailand, UK, and USA (Fig. 13C–E; Supplementary data S4).

## Discussion

The molecular mechanisms of resistance and virulence dissemination in clinical *K. pneumoniae* circulating in two referral hospitals in South Africa were characterised and found to be richly endowed with diverse determinants of resistance, virulence and mobile-genetic elements. Notably, the isolates were MDR to several clinically important antibiotics except for reserved ones such as the carbapenems, colistin and tigecycline. The presence of these MDR strains in specimens from this diverse patient demographics in two important referral hospitals make this a very worrying finding. Particularly, same STs were identified in both referral hospitals, suggesting their circulation in both health centres. As expected, the strains’ phenotypic resistance characteristics correlated with known genetic mediators of resistance except for amikacin, nalidixic acid, minocycline, and fosfomycin for which the presence of resistance genes such as *aac, aad* and *aph, tet(A/D/J*) and *fosA* led to no phenotypic resistance. Whereas we could not undertake expression analyses to determine the expression state of these genes, we suspect that the lack of resistance in their presence could be due to little or no expression.

The presence of the *bla*_CTX-M_, *bla*_TEM_, *bla*_OXA_, and *bla*_SHV_ ESBL genes in similar genetic contexts have been described previously in same and different species in South African and international isolates^7,8,12,19,20,31–36^. The presence of these ARGs within the same genetic context and on the same plasmid replicons across same and different species around the globe strongly suggests the clonal and plasmid-mediated spread of these ARGs. Specifically, IS*Ec9* and IncF plasmids have been shown to mobilize and facilitate the global spread of *bla*_CTX-M-15_, alongside *aac(6*′*)Ib-cr, bla*_*OXA-10*_ *and bla*_TEM_, across species^6,7,12,37^. Thus, it is not surprising to have the IS*Ec9* and IncF plasmid replicons dominating in these strains that all harboured *bla*_CTX-M-15_ alongside *aac(6*′*)Ib-cr, bla*_*OXA-10*_ *and bla*_TEM._ Interestingly, the same genetic context around the *aac(6*′*)Ib-cr, bla*_*OXA-10*_ *and bla*_TEM_ genes were also observed in *E. coli* strains from the same hospitals, suggesting plasmid-mediated circulation of these genes within these academic hospitals^12^.

*bla*_CTX-M-15_ being present in all the isolates is worth noting, but is not new as an earlier study also found this gene in all the Enterobacteriaceae species studied^7^. Other studies from South Africa have described the presence of the *bla*_CTX-M-15_ gene in *E. coli* isolates and more recently, in *K. pneumoniae*^7,12,33^. We also report a higher prevalence (87.5%) of OXA β–lactamase genes in these *K. pneumoniae* isolates as well as *bla*_SHV_ in 83.3% of isolates, confirming that this supposedly chromosomally encoded gene, is not universally found in *K. pneumoniae* species^38^. Interestingly, four isolates also contained the narrow spectrum, chromosomally encoded *bla*_LEN_ gene, comprising of *bla*_LEN9_ and *bla*_LEN12_, which are rare in South Africa and Africa although it has been previously described in Kenya^39^. The *bla*_LEN_ β-lactamase gene was first identified by Arakawa^30^.

The global dominance of the IncF plasmid, a MGE associated with HGT, is thus herein confirmed^6,7,40^. As well, an association between IncF replicons and multi-drug resistance (MDR) was observed, as reported globally^6,10,11^. The presence of several plasmids in which integrons that capture cassette genes are located has been shown to cause MDR^6,10,11^. The rich repertoire of ARGs in these isolates suggest the presence of one or multiple plasmids, corroborated by the plasmid replicons (Supplementary Table 1).

The integrons identified herein contained diverse gene cassettes, which are novel in the South African context. Particularly, the *dfrA* and *aadA* gene cassettes corroborates the global spread of these MGEs^41,42^. Moreover, *aadA1* types, *aadA1b* and *aadA1a*, which are different from results reported in *K. pneumoniae* from Korea where *aadA2* types were more frequently identified, were observed. These differences signify subtle changes in genetic composition at a local level^11,13,31^. Overall, 20 gene cassettes in 14 different cassette arrays, which have not been described previously, were observed. Significantly, the most prevalent cassette arrays viz., *dfrA*1b-*aadA*1a and *dfrA*1b-*aadA*1b, differed from those described by Partridge *et al*., which included *aadA1a, aadA2* and *aadB* cassettes^11,13^.

Integron In369, which captured the *dfr*A1b – *aad*A1b cassette array was identified for the first time in South African isolates. This integron and cassette array were also reported in a Portuguese environmental study^43^. In the current study, this integron was more frequently associated with ST1552 and ST152 but was also found in two ST101 isolates, suggesting its broad host range and promiscuity. Interestingly, integron 27, capturing the *dfr*A12-*gcu*F–*aad*A2 cassette array was only identified in ST234 isolates. Integron 191 was however identified in four different STs suggesting a diversity of clones with this MGE. The dominance of the class 1 integron in this study is also consistent with genomes found in Africa, Europe and South America^8,31,41,44^. Two novel integrons identified had the *aad*A16 cassette variant in K021 as well as *dfr*30b and *dfr*A14b cassette variants in K145, confirming the ongoing evolutionary processes in these genomes resulting in the diversity of gene cassettes in *K. pneumoniae*.

Tn*3* transposons, which bracketed *bla*_CTX-M-15_, *bla*_TEM-1B_, *aac(6*′*)-Ib-cr*, _*6*′*)-r*_, *aph(6)-Id, aph(3*′*)* and *sul2* with myriad ISs in the isolates (Figs. 2–7), commonly encode resistance to β-lactams^45–48^; this synteny and genetic environment were also observed in *E. coli* from the same hospitals^12^. This rich diversity of transposons and ISs obviously contribute to the genome plasticity, ARGs composition and HGT of ARGs within and across the strains^6,8,10^. None of the isolates carried Tn21, which was shown to carry multiple resistance genes in a Kenyan study, and was associated with the transfer of antimicrobial resistance in these isolates^44^.

Our findings suggest plasmid and chromosomally mediated quinolone resistance genes in the clinical *K. pneumoniae* isolates. The simultaneous presence of *qnr, oqx* and *aac(6*″*)-lb-cr* PMQR genes in these quinolone-resistant isolates and the dominance of the latter is consistent with the literature^49–53^. This is the second report of the *oqxAB* gene being found in Africa, although previously described in carbapenemase-producing *Enterobacteriaceae*^51,54^. Significantly, we did not detect the *qep* efflux pump gene which is not frequently reported^55,56^. In this study, 90% of isolates contained the *aac(6*′*)-lb-cr* gene, which is of higher prevalence than studies reported from Spain, Uruguay and Sweden^53,57–59^, which could be explained by the localised spread of related sequence types carrying this gene. Mutations such as S**83**A and D**87**A/G in *gyrA*, D**553**V and Q/L**657**G/M in *gyrB* and S**80**I and N**304**S in *parC* were also found in *K. pneumoniae* and other Enterobacteriaceae in Durban, South Africa^51^. Coupled with the PMQR genes, these mutations underlies the resistance to fluoroquinolones to some of the strains (Table 3), albeit no resistance was expressed towards norfloxacin^60^.

Hospital-acquired Gram-negative infections are usually caused by multi-drug resistant organisms, limiting options available for treating such patients^1–3,61^. Herein, these isolates from hospitalised patients simultaneously contained ESBL β-lactamase genes and PMQR genes, as well as co-resistance to other antibiotic classes, implying that significant antibiotic use causes resistance, co-selection of resistance genes and more significantly, HGT (horizontal gene transfer)^3,54,62^. These genes have been shown to be co-transmitted on plasmids and other MGEs^6,11^.

All the isolates in this study were trimethoprim-resistant, although trimethoprim-sulfamethoxazole is no longer recommended for treatment of outpatient conditions such as urinary tract infections in South Africa and internationally because of the high prevalence of resistance^63–65^. Similarly, fosfomycin, used in the outpatient setting for urinary tract infections may not be efficacious since most isolates had the *fos*A gene, a finding only recently described in *Enterobacteriaceae* in South Africa^7,64,65^. The presence of chromosomal colistin resistance in nine out of the 11 ESBL-containing isolates, with an MIC ≥ 4 g/mL, is of concern (Table 3). However, we were unable to confirm these with the broth microdilution, which is the recommended method for colistin resistance determination^66^. No *mcr* gene was found, suggesting that these were vertically acquired or engendered *de novo*.

The diversity and complexity of the virulome and capsule types identified among the strains are concerning as they are implicated in virulence. Fortunately, no hypervirulence genes were found albeit capsule type K2, identified in some strains (Figs. 9–12), are associated with increased virulence and resistance^67–69^. It is interesting to note that the K2 serotype was also identified in a ST14 strain (K118) in this study as was reported in China recently^69^. The highly clone-specific nature of the O and K capsule types suggest their conserved nature within the genome, contrary to the virulence genes, which differed even within clones (Figs. 3 and 4). The diversity of the virulence genes within clones from the same hospital setting suggest that they were mostly acquired horizontally rather than vertically and could be associated with plasmids. We were unfortunately unable to determine their mobility and presence on plasmids. Further, no association could be established between the specimen sources and the virulome or capsule types. K094 presents a very interesting observation in that it contained no virulence gene or capsules, had no mutations in genes conferring resistance to fluoroquinolones and colistin, contained very few resistance genes (n = 8) and was phylogenetically distant from all STs and clades (Figs. 3–5).

The diversity of sequence types identified in these MDR *K. pneumoniae* isolates is consistent with findings reported from South, Central and North America, Europe, Asia and North Africa^70–74^. While ST152 and ST1552 were dominant in this study, and related to Chinese, Ghanaian and Thai genomes, they are not often described in the literature, compared to ST238, an ST responsible for many CRE outbreaks globally^6,75,76^. The only globally reported ST identified was ST15, confirming the global diversity of this clonal group that was identified in 15% of *K. pneumoniae* CRE isolates in an international multicentre study encompassing Morocco, Cameroon, Senegal, Madagascar and Vietnam^77^. ST152 has also been reported in Durban^14^, as a major clone in Cuba^77^ and as a major career of NDM-1 in Saudi Arabia^78^. However, ST147 and ST258, which are global STs associated with antimicrobial resistance^79–81^, were not detected in these isolates. Most sequence types had global spread, being more frequently related to Ghanaian, Thai and Chinese isolates. Four sequence types, viz., ST1552, ST234, ST1414 and ST152 also demonstrated local spread. Local and international outbreaks, in both South Africa and abroad, were also observed in the trees under distinct clades.

An analysis of the molecular epidemiology of these isolates confirmed the international dissemination of specific STs between South Africa and the world. Clonal similarity of isolates from South Africa, Thailand, Nigeria and China was evident although there was a diversity of sequence types associated with other countries. There was also similarity in clones within South Africa; specifically, between Durban and Pretoria. The resolution power of whole-genome sequencing over MLST is demonstrated herein by the clustering of different STs on the same branch and clade, supporting the need to shift to genomic epidemiology for better epidemiological surveillance and infection control.

## Conclusion

The burden of ARGs and virulence genes in these isolates from hospitalised patients in two major referral hospitals within Pretoria confirm the global threat of ABR, mediated by MGEs. The findings demonstrate the centrality of MGEs in defining the resistome of MDR strains. Phylogenetic analysis confirmed this global spread including evolutionary relationships of the different STs. IncF plasmid replicons and class1 integrons, both of which have been globally reported, were also dominant in these isolates. Significantly, two novel integrons were identified. The presence of the rare chromosomal *bla*_LEN_ gene in four isolates is also notable.

### Ethics

Ethical approval was provided by the Human Research Ethics Committee of the University of Witwatersrand (Ref M1710100). All protocols and consent forms were executed according to the agreed ethical approval terms and conditions. All clinical samples were obtained from a reference laboratory and not directly from patients, who agreed to our using their specimens for this research. The guidelines stated by the Declaration of Helsinki for involving human participants were followed in the study.

## Supplementary information

Supplementary Table S1.

Supplementary Table S2.

Supplementary Data S3.

Supplementary Data S4.
